# P04.87. Are people with known coronary risk factors more likely to use dietary supplements?

**DOI:** 10.1186/1472-6882-12-S1-P357

**Published:** 2012-06-12

**Authors:** D Zamora, K Faurot, L Young, S Gaylord

**Affiliations:** 1 UNC School of Medicine, Chapel Hill, USA

## Purpose

Use of dietary supplements by individuals are thought to be associated with health-seeking behaviors. The aim of this study was to examine the patterns of dietary supplement (DS) use among individuals with coronary risk factors (CRF): prediabetes/diabetes (Type 2), hypertension, elevated cholesterol, smoking, overweight/obesity, and physical inactivity. We hypothesized that people with known risk factors would be more likely to use DS.

## Methods

We assessed the prevalence of DS use among respondents to the alternative health supplement of the 2007 National Health Interview Survey (NHIS), a national multistage probability sample of non-institutionalized U.S. residents (n=23,388). All measures were self-reported. DS include herbal/specialty products as well as vitamin and mineral supplements taken during the last 30 days. We calculated prevalence odds ratios (POR) for DS use using weighted logistic regression models adjusted for age, gender, education, race/ethnicity, and access to care (insurance), as well as smoking and physical inactivity where appropriate.

## Results

About 13% of the population reported taking any DS during the last month and 95% reported at least one CRF. Individuals with 4-6 CRFs had 1.15 times the odds of DS use compared with those with 0-1 CRF (POR: 1.15, p=0.01). However, the association between individual CRFs and DS use varied widely. Individuals with prediabetes, elevated cholesterol, and smoking were significantly more likely to use DS (POR=1.38, 1.30, and 1.16, respectively). In contrast, those with diabetes, overweight/obesity, and inactivity were significantly less likely to use DS (POR= 0.87, 0.90, and 0.50, respectively). DS use was not associated with hypertension.

## Conclusion

We found that some risk factors were associated with higher use of DS, while others were associated with lower use. It may be that being overweight and inactive are not recognized as important risk factors, while smoking and elevated cholesterol are. Our findings of divergent associations for diabetes and prediabetes are intriguing, possibly reflecting health-seeking behavior among prediabetics while less present amongst diebetic individuals.

